# Supplementary material for: The Risk of Metabolic Dysfunction-Associated Steatotic Liver Disease in Moderate-to-Severe Psoriasis: A Systematic Review and Meta-Analysis
Source: J Clin Med. 2025 Feb 19;14(4):1374. doi: 10.3390/jcm14041374 (PMC11855964; doi:10.3390/jcm14041374)
Supplement: Supplementary file 1 [file jcm-14-01374-s001.zip › TableS1-S7.pdf]

PRISMA 2020 Checklist

Table S1: PRISMA checklist

Section and Topic	Item #	Checklist item	Location where item is reported
**TITLE**			
Title	1	Identify the report as a systematic review.	1
**ABSTRACT**			
Abstract	2	See the PRISMA 2020 for Abstracts checklist.	3
**INTRODUCTION**			
Rationale	3	Describe the rationale for the review in the context of existing knowledge.	4-5
Objectives	4	Provide an explicit statement of the objective(s) or question(s) the review addresses.	5
**METHODS**			
Eligibility criteria	5	Specify the inclusion and exclusion criteria for the review and how studies were grouped for the syntheses.	6-7
Information sources	6	Specify all databases, registers, websites, organisations, reference lists and other sources searched or consulted to identify studies. Specify the date when each source was last searched or consulted.	6
Search strategy	7	Present the full search strategies for all databases, registers and websites, including any filters and limits used.	6, Table S2
Selection process	8	Specify the methods used to decide whether a study met the inclusion criteria of the review, including how many reviewers screened each record and each report retrieved, whether they worked independently, and if applicable, details of automation tools used in the process.	6-7
Data collection process	9	Specify the methods used to collect data from reports, including how many reviewers collected data from each report, whether they worked independently, any processes for obtaining or confirming data from study investigators, and if applicable, details of automation tools used in the process.	6-7
Data items	10a	List and define all outcomes for which data were sought. Specify whether all results that were compatible with each outcome domain in each study were sought (e.g. for all measures, time points, analyses), and if not, the methods used to decide which results to collect.	6-7
	10b	List and define all other variables for which data were sought (e.g. participant and intervention characteristics, funding sources). Describe any assumptions made about any missing or unclear information	6-7
Study risk of bias assessment	11	Specify the methods used to assess risk of bias in the included studies, including details of the tool(s) used, how many reviewers assessed each study and whether they worked independently, and if applicable, details of automation tools used in the process.	8
Effect measures	12	Specify for each outcome the effect measure(s) (e.g. risk ratio, mean difference) used in the synthesis or presentation of results.	8
Synthesis methods	13a	Describe the processes used to decide which studies were eligible for each synthesis (e.g. tabulating the study intervention characteristics and comparing against the planned groups for each synthesis (item #5)).	6-7
	13b	Describe any methods required to prepare the data for presentation or synthesis, such as handling of missing summary statistics, or data conversions.	6-8
	13c	Describe any methods used to tabulate or visually display results of individual studies and syntheses.	6-8, Figure 1
	13d	Describe any methods used to synthesize results and provide a rationale for the choice(s). If meta-analysis was performed, describe the model(s), method(s) to identify the presence and extent of statistical heterogeneity, and software package(s) used.	6-8
	13e	Describe any methods used to explore possible causes of heterogeneity among study results (e.g. subgroup analysis, meta-regression).	8
	13f	Describe any sensitivity analyses conducted to assess robustness of the synthesized results.	8
Reporting bias assessment	14	Describe any methods used to assess risk of bias due to missing results in a synthesis (arising from reporting biases).	7-8

PRISMA 2020 Checklist

Section and Topic	Item #	Checklist item	Location where item is reported
Certainty assessment	15	Describe any methods used to assess certainty (or confidence) in the body of evidence for an outcome.	8
**RESULTS**			
Study selection	16a	Describe the results of the search and selection process, from the number of records identified in the search to the number of studies included in the review, ideally using a flow diagram.	9 and Figure1
	16b	Cite studies that might appear to meet the inclusion criteria, but which were excluded, and explain why they were excluded.	9
Study characteristics	17	Cite each included study and present its characteristics.	9-10 and Table 1
Risk of bias in studies	18	Present assessments of risk of bias for each included study.	11, Table S6
Results of individual studies	19	For all outcomes, present, for each study: (a) summary statistics for each group (where appropriate) and (b) an effect estimate and its precision (e.g. confidence/credible interval), ideally using structured tables or plots.	9-13, Figure 2, Figure S1-S5
Results of syntheses	20a	For each synthesis, briefly summarise the characteristics and risk of bias among contributing studies.	9-13, Table 1, S6
	20b	Present results of all statistical syntheses conducted. If meta-analysis was done, present for each the summary estimate and its precision (e.g. confidence/credible interval) and measures of statistical heterogeneity. If comparing groups, describe the direction of the effect.	9-13, Figure 2, Figure S1-S5
	20c	Present results of all investigations of possible causes of heterogeneity among study results.	13
	20d	Present results of all sensitivity analyses conducted to assess the robustness of the synthesized results.	13, Figure s1
Reporting biases	21	Present assessments of risk of bias due to missing results (arising from reporting biases) for each synthesis assessed.	11
Certainty of evidence	22	Present assessments of certainty (or confidence) in the body of evidence for each outcome assessed.	11-12, Table S7
**DISCUSSION**			
Discussion	23a	Provide a general interpretation of the results in the context of other evidence.	14
	23b	Discuss any limitations of the evidence included in the review.	16
	23c	Discuss any limitations of the review processes used.	16
	23d	Discuss implications of the results for practice, policy, and future research.	15
**OTHER INFORMATION**			
Registration and protocol	24a	Provide registration information for the review, including register name and registration number, or state that the review was not registered.	6
	24b	Indicate where the review protocol can be accessed, or state that a protocol was not prepared.	6
	24c	Describe and explain any amendments to information provided at registration or in the protocol.	6
Support	25	Describe sources of financial or non-financial support for the review, and the role of the funders or sponsors in the review.	23
Competing interests	26	Declare any competing interests of review authors.	23
Availability of data, code and other materials	27	Report which of the following are publicly available and where they can be found: template data collection forms; data extracted from included studies; data used for all analyses; analytic code; any other materials used in the review.	23

PRISMA 2020 Checklist

From: Page MJ, McKenzie JE, Bossuyt PM, Boutron I, Hoffmann TC, Mulrow CD, et al. The PRISMA 2020 statement: an updated guideline for reporting systematic reviews. BMJ 2021;372:n71. doi: 10.1136/bmj.n71
For more information, visit: http://www.prisma-statement.org/

Table S2: Search strategy

Database: Ovid MEDLINE

1. psoriasis.mp. OR exp Psoriasis/
2. obesity.mp. or exp Obesity/
3. overweight.mp. or exp Overweight/
4. body mass index.mp. or exp Body Mass Index/
5. obese.mp.
6. bmi.mp.
7. or/2-6
8. metabolic syndrome.mp. or exp Metabolic Syndrome/
9. blood pressure$.mp. or exp Blood Pressure/
10. hypertension.mp or exp Hypertension/
11. hypertensive$.mp
12. systolic$.mp
13. diastolic$.mp
14. or/9-13
15. nonalcoholic fatty liver.mp. or exp Non-alcoholic Fatty Liver Disease/
16. non-alcoholic fatty liver.mp.
17. nonalcoholic steatohepatitis.mp.
18. non-alcoholic steatohepatitis.mp.
19. metabolic dysfunction-associated steatohepatitis.mp.
20. metabolic dysfunction associated steatotic liver disease.mp.
21. metabolic dysfunction-associated steatotic liver disease.mp.
22. NASH.mp.
23. NAFLD.mp.
24. NAFL.mp.
25. MASH.mp.
26. MASLD.mp.
27. fatty liver.mp. or exp Fatty Liver/
28. nonalcoholic liver disease.mp.
29. non-alcoholic liver disease.mp.
30. or/15-29
31. diabetes mellitus.mp. or exp Diabetes Mellitus/
32. diabetes.mp.
33. exp Diabetes Mellitus, Type 1/ or exp Diabetes Mellitus, Type 2/
34. hyperglycemia.mp. or exp Hyperglycemia/
35. hyperglycaemia.mp.
36. exp Insulin Resistance/
37. or/31-36
38. dyslipid$.mp. or exp Dyslipidemia/

39. hyperlipid$.mp. or exp Hyperlipidemia/
40. hypercholesterol$ or exp Hypercholesterolemia/
41. hypertriglyceridemia.mp. or exp Hypertriglyceridemia/
42. or/38-41
43. coronary artery disease.mp. or exp Coronary Artery Disease/
44. coronary heart disease.mp.
45. ischemic heart disease.mp. or exp Ischemic Heart Disease/
46. coronary artery obstruction.mp. or exp Coronary Artery Obstruction/
47. coronary artery thrombosis.mp. or exp Coronary Artery Thrombosis/
48. coronary artery stenosis.mp.
49. heart infarction.mp. or exp Heart Infarction/
50. myocardial infarct$.mp. or exp Myocardial Infarction/
51. myocardial ischemia.mp. or exp Myocardial Ischemia/
52. angina.mp. or exp Angina Pectoris/
53. unstable angina.mp. or exp Unstable Angina Pectoris/
54. chronic coronary syndrome.mp.
55. acute coronary syndrome.mp. or exp Acute Coronary Syndrome/
56. or/43-55
57. stroke.mp. or exp Stroke/
58. cerebrovascular disease.mp. or exp Cerebrovascular Disorders/
59. cerebrovascular accident.mp.
60. brain infarct$.mp. or exp Brain Infarction/
61. cerebral infarct$.mp. or exp Cerebral Infarction/
62. brain ischemia.mp. or exp Brain Ischemia/
63. cerebral ischemia.mp.
64. or/57-63
65. 1 and (7 or 8 or 14 or 30 or 37 or 42 or 56 or 64)

Database: EMBASE

1. 'psoriasis' OR 'Psoriasis'/exp
2. 'obesity' or 'Obesity'/exp
3. 'overweight' or 'Overweight'/exp
4. 'body mass index' or 'Body Mass Index'/exp
5. 'obese'
6. 'bmi'
7. #2 OR #3 OR #4 OR #5 OR #6
8. 'metabolic syndrome*' or 'Metabolic Syndrome'/exp
9. 'blood pressure*' or 'Blood Pressure'/exp
10. 'hypertension' or 'Hypertension'/exp
11. 'hypertensive*'
12. 'systolic*'
13. 'diastolic*'
14. #9 OR #10 OR #11 OR #12 OR #13
15. 'nonalcoholic fatty liver' or 'Non-alcoholic Fatty Liver Disease'/exp
16. 'non-alcoholic fatty liver'
17. 'nonalcoholic steatohepatitis'
18. 'non-alcoholic steatohepatitis'
19. 'metabolic dysfunction-associated steatohepatitis'
20. 'NASH'
21. 'NAFLD'
22. 'NAFL'
23. 'MASH'
24. 'fatty liver' or 'Fatty Liver'/exp
25. 'nonalcoholic liver disease'
26. 'non-alcoholic liver disease'
27. #15 OR #16 OR #17 OR #18 OR #19 OR #20 OR #21 OR #22 OR #23 OR #24 OR #25 OR #26
28. 'diabetes mellitus' or 'Diabetes Mellitus'/exp
29. 'diabetes'
30. 'Diabetes Mellitus, Type 1'/exp or 'Diabetes Mellitus, Type 2'/exp
31. 'hyperglycemia' or 'Hyperglycemia'/exp
32. 'hyperglycaemia'
33. 'Insulin Resistance'/exp
34. #28 OR #29 OR #30 OR #31 OR #32 OR #33
35. 'dyslipid*' or 'Dyslipidemia'/exp
36. 'hyperlipid*' or 'Hyperlipidemia'/exp
37. 'hypercholesterol*' or 'Hypercholesterolemia'/exp
38. 'hypertriglyceridemia' or 'Hypertriglyceridemia'/exp

39. #35 OR #36 OR #37 OR #38
40. 'coronary artery disease' or 'Coronary Artery Disease'/exp
41. 'coronary heart disease'
42. 'ischemic heart disease' or 'Ischemic Heart Disease'/exp
43. 'coronary artery obstruction' or 'Coronary Artery Obstruction'/exp
44. 'coronary artery thrombosis' or 'Coronary Artery Thrombosis'/exp
45. 'coronary artery stenosis'
46. 'heart infarction' or 'Heart Infarction'/exp
47. 'myocardial infarct*' or 'Myocardial Infarction'/exp
48. 'myocardial ischemia' or 'Myocardial Ischemia'/exp
49. 'angina' or 'Angina Pectoris'/exp
50. 'unstable angina' or 'Unstable Angina Pectoris'/exp
51. 'chronic coronary syndrome'
52. 'acute coronary syndrome' or 'Acute Coronary Syndrome'/exp
53. #40 OR #41 OR #42 OR #43 OR #44 OR #45 OR #46 OR #47 OR #48 OR #49 OR #50 OR #51 OR #52
54. 'stroke' or 'Stroke'/exp
55. 'cerebrovascular disease' or 'Cerebrovascular Disorders'/exp
56. 'cerebrovascular accident'
57. 'brain infarct*' or 'Brain Infarction'/exp
58. 'cerebral infarct*' or 'Cerebral Infarction'/exp
59. 'brain ischemia' or 'Brain Ischemia'/exp
60. 'cerebral ischemia'
61. #54 OR #55 OR #56 OR #57 OR #58 OR #59 OR #60
62. #1 AND (#7 OR #8 OR #14 OR #27 OR #34 OR #39 OR #53 OR #61)

Database: ClinicalTrials.gov

("non alcoholic fatty liver" OR "nonalcoholic fatty liver" OR "non-alcoholic fatty liver" OR "nonalcoholic steatohepatitis" OR "non-alcoholic steatohepatitis" OR "metabolic dysfunction-associated steatohepatitis" OR "NASH" OR "NAFLD" OR "NAFL" OR "MASH" OR "fatty liver" OR "Fatty Liver" OR "nonalcoholic liver disease" OR "non-alcoholic liver disease" OR “MASLD” OR “Metabolic Dysfunction-Associated Steatotic Liver Disease” OR “Metabolic Dysfunction Associated Steatotic Liver Disease”) AND ("psoriasis")

Database: OSF

("non alcoholic fatty liver" OR "nonalcoholic fatty liver" OR "non-alcoholic fatty liver" OR "nonalcoholic steatohepatitis" OR "non-alcoholic steatohepatitis" OR "metabolic dysfunction-associated steatohepatitis" OR "NASH" OR "NAFLD" OR "NAFL" OR "MASH" OR "fatty liver" OR "Fatty Liver" OR "nonalcoholic liver disease" OR "non-alcoholic liver disease" OR “MASLD” OR “Metabolic Dysfunction-Associated Steatotic Liver Disease” OR “Metabolic Dysfunction Associated Steatotic Liver Disease”) AND ("psoriasis")

medRxiv

1. "non alcoholic fatty liver"
2. "nonalcoholic fatty liver"
3. "non-alcoholic fatty liver"
4. "nonalcoholic steatohepatitis"
5. “non-alcoholic steatohepatitis”
6. "metabolic dysfunction-associated steatohepatitis"
7. "NASH"
8. "NAFLD"
9. "NAFL"
10. "MASH"
11. "fatty liver"
12. "Fatty Liver"
13. "nonalcoholic liver disease"
14. "non-alcoholic liver disease"
15. “MASLD”
16. “Metabolic Dysfunction-Associated Steatotic Liver Disease”
17. “Metabolic Dysfunction Associated Steatotic Liver Disease”
18. "psoriasis"
19. #1 OR #2 OR #3 OR #4 OR #5 OR #6 OR #7 OR #8 OR #9 OR #10 OR #11 OR #12 OR #13 OR #14 OR #15 OR #16 OR #17 OR #18
20. #18 AND #19

Table S3: Diagnostic criteria for psoriasis in the eligible articles

Author, year	Diagnostic criteria
Awosika, 2018 [28]	NA
Basireddy, 2016 [30]	Diagnostic criteria was pertained to the physical examination by the dermatologist.
Chavhan, 2023 [25]	Diagnostic criteria was primarily based on signs and symptoms.
Gisondi, 2009 [9]	The dermatologist attributed the diagnosis to the relevant physical examination.
Mahajan, 2022 [29]	Diagnosis was mainly related to the pertinent signs and symptoms.
Näslund-Koch, 2022 [24]	Diagnosis was determined from ICD-8 code (696.09, 696.10, 696.19) and ICD-10 code L40 in hospitalized patients.
Panjiyar, 2023 [26]	NA
Yadav, 2023 [27]	Diagnosis was determined using relative clinical findings.

Abbreviations: ICD, International Classification of Diseases; NA, not applicable

Table S4: Criteria for severity assessment in moderate-to-severe psoriasis in the eligible articles

Author, year	Severity assessment
Awosika, 2018 [28]	Patients with psoriasis were graded as moderate to severe in PASI>10 and BSA >10%.
Basireddy, 2016 [30]	Patients with moderate to severe psoriasis were classified from PASI>10.
Chavhan, 2023 [25]	Patients with severe psoriasis were identified based on a PASI score of more than 10.
Gisondi, 2009 [9]	Patients with moderate to severe psoriasis were clarified from at least 10 of PASI score.
Mahajan, 2022 [29]	Patients with moderate psoriasis were defined by a PASI score ranging from 6 to 12 or a BSA involvement between 11% and 20%. Severe psoriasis was defined by a PASI score exceeding 12 or a BSA involvement greater than 20%.
Näslund-Koch, 2022 [24]	Hospitalized patients who had ICD-8 code (696.09, 696.10, 696.19) and ICD-10 code L40, were graded as moderate to severe psoriasis.
Panjiyar, 2023 [26]	NA
Yadav, 2023 [27]	Patients with severe psoriasis was graded from PASI score of ≥10 or BSA involvement of≥10 %.

List of abbreviations: BSA, Body Surface Area; ICD, International Classification of Diseases; PASI, Psoriasis Area Severity Index.

Table S5: Diagnostic criteria for metabolic dysfunction-associated steatotic liver disease in the included articles

Author, year	Diagnostic criteria
Awosika, 2018 [28]	Utilizing ultrasonography for diagnosis, characterized by heightened attenuation of the liver with an obscure diaphragm, enhanced echogenicity of the liver compared with renal parenchyma, or diminished distinctions of intensity in periportal region and vesicular wall. Patients who have risks of metabolic syndrome were excluded.
Basireddy, 2016 [30]	NA
Chavhan, 2023 [25]	Diagnostic criteria were primarily based on ultrasound representing liver steatosis, transaminitis (ALT > 30 U/L in men, > 19U/L in women), triglyceridemia (more than 150 mg/dL). The other causes, including alcohol, viral hepatitis, drugs, congenital liver disease, history of cancer, were excluded.
Gisondi, 2009 [9]	Diagnostic criteria were determined based on relevant findings of ultrasound, including enhancing echogenicity compared with renal parenchyma, attenuation of liver, blurred resolution of liver. We excluded potential causes such as cancer, alcohol consumption, liver cirrhosis, viral hepatitis, autoimmune hepatitis, or drugs.
Mahajan, 2022 [29]	Ultrasonographic findings, including enhancing hyperechogenicity of the liver related to renal parenchyma, attenuation of the liver, and blurred resolution of the liver, were diagnostic criteria. The hepatic steatosis was assessed via fibroscan through controlled attenuation parameter (CAP). We excluded potential causes such as cancer, alcohol consumption, liver disease, or drugs.
Näslund-Koch, 2022 [24]	Patients who had ICD-10 codes (K75.9, K76.0 and K76.9) were retrieved.
Panjiyar, 2023 [26]	Diagnostic criteria were primarily based on ultrasound representing enhancing echogenicity compared with renal parenchyma, obscuration of hepatic veins and diaphragm, transaminitis (ALT > 26 U/L in boys, > 22U/L in girls). Other liver diseases were excluded.
Yadav, 2023 [27]	Using ultrasound, LFT, and fibroscan for diagnosis while eliminating possible causes of NAFLD, such as alcohol drinking, viral hepatitis, Wilson's disease, primary biliary cirrhosis, hepatocellular carcinoma, risks of metabolic syndrome, or drugs

Abbreviations: ALT, Alanine aminotransferase; CAP, controlled attenuation parameter; ICD, International Classification of Diseases; LFT, Liver Function Test; NAFLD, non-alcoholic fatty liver disease; NA, not applicable

Table S6: The quality assessment in each included article

Author, year	Selection	Comparability	Outcome	Total score
Awosika, 2018 [28]	4	1	3	8
Basireddy, 2016 [30]	4	2	2	8
Chavhan, 2023 [25]	5	1	3	9
Gisondi, 2009 [9]	4	2	3	9
Mahajan, 2022 [29]	4	2	3	9
Näslund-Koch, 2022 [24]	5	2	3	10
Panjiyar, 2023 [26]	5	1	2	8
Yadav, 2023 [27]	5	2	2	9

Table S7: GRADE assessment for metabolic dysfunction-associated steatotic liver disease outcome in moderate-to-severe psoriasis patients

Outcome	No. of studies	Result [OR (95%CI)]	Risk of bias	Inconsistency	Indirectness	Imprecision	Publication bias	Confidence
MASLD	8	4.10 (2.17,7.77)	Serious	Serious	Not serious	Not serious	None	⊕⊕◯◯ low

List of abbreviations: CI, confidence interval; MASLD, metabolic dysfunction-associated steatotic liver disease; OR, odds ratio
