# Supplementary figures and images for: The Risk of Metabolic Dysfunction-Associated Steatotic Liver Disease in Moderate-to-Severe Psoriasis: A Systematic Review and Meta-Analysis
Source: J Clin Med. 2025 Feb 19;14(4):1374. doi: 10.3390/jcm14041374 (PMC11855964; doi:10.3390/jcm14041374)

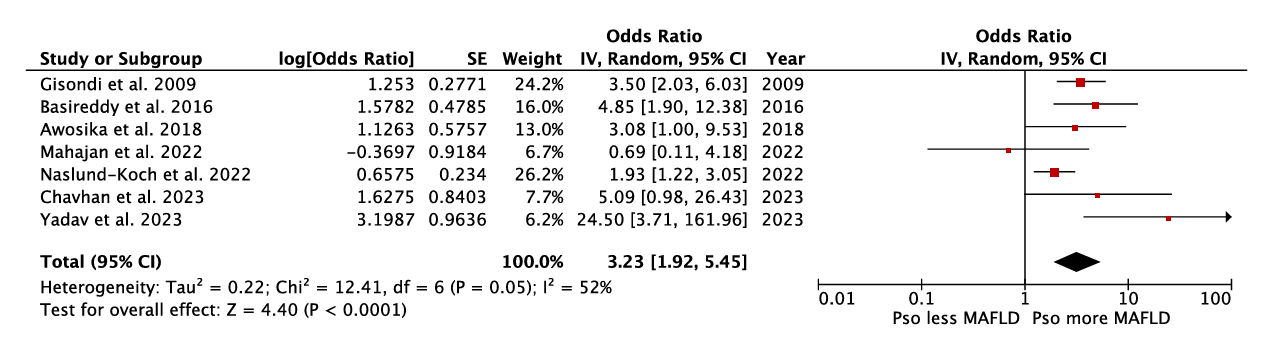

Supplement: Supplementary file 1 [file jcm-14-01374-s001.zip › Figure S1.PNG]

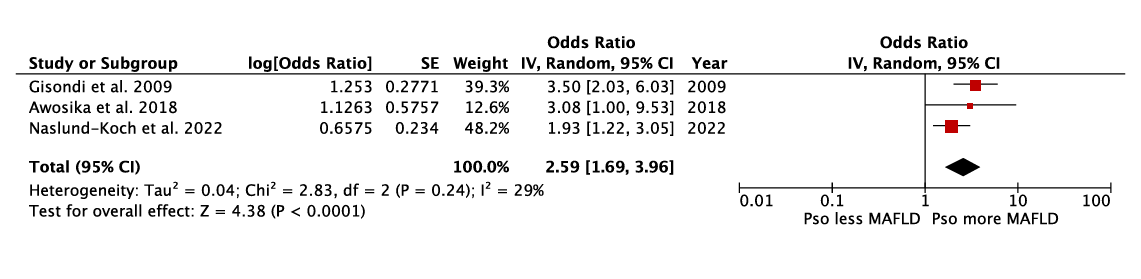

Supplement: Supplementary file 1 [file jcm-14-01374-s001.zip › Figure S2.PNG]

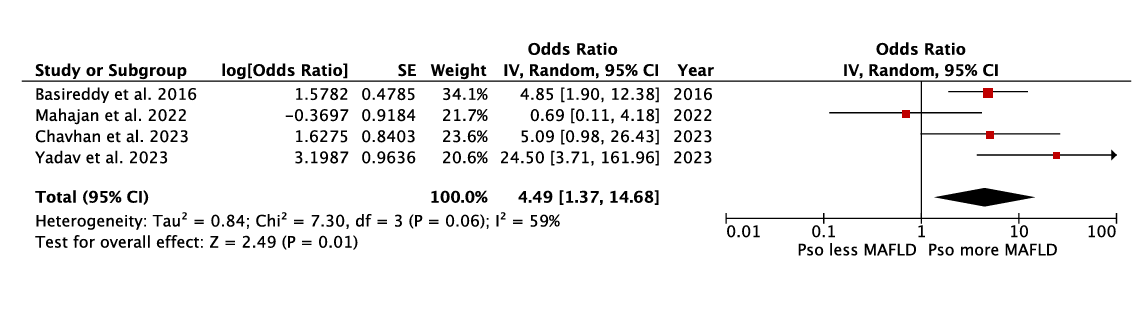

Supplement: Supplementary file 1 [file jcm-14-01374-s001.zip › Figure S3.PNG]

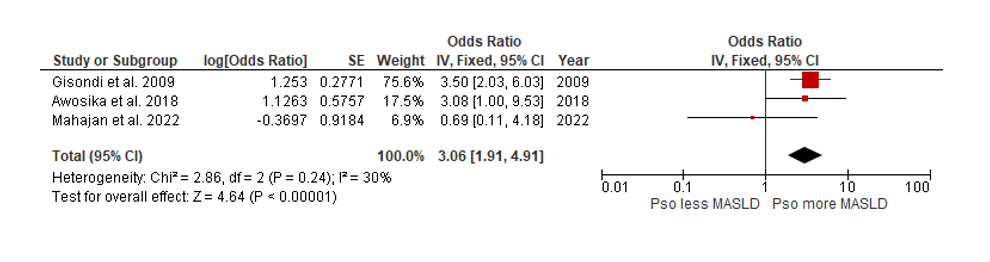

Supplement: Supplementary file 1 [file jcm-14-01374-s001.zip › Figure S4.PNG]

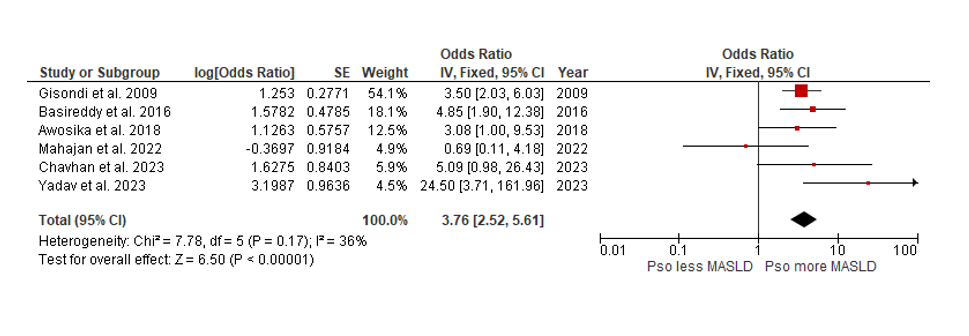

Supplement: Supplementary file 1 [file jcm-14-01374-s001.zip › Figure S5.PNG]
